# Near-Field Beamforming Algorithms for UAVs

**DOI:** 10.3390/s23136172

**Published:** 2023-07-05

**Authors:** Yinan Zhang, Guangxue Wang, Shirui Peng, Yi Leng, Guowen Yu, Bingqie Wang

**Affiliations:** Department of Information Countermeasures, Air Force Early Warning Academy, Wuhan 430010, China

**Keywords:** UAVs, near-field beamforming, phase error compensation, Kalman Filter, Extended Kalman Filter, Unscented Kalman Filter

## Abstract

This study presents three distributed beamforming algorithms to address the challenges of positioning and signal phase errors in unmanned aerial vehicle (UAV) arrays that hinder effective beamforming. Firstly, the array’s received signal phase error model was analyzed under near-field conditions. In the absence of navigation data, a beamforming algorithm based on the Extended Kalman Filter (EKF) was proposed. In cases where navigation data were available, Taylor expansion was utilized to simplify the model, the non-Gaussian noise of the compensated received signal phase was approximated to Gaussian noise, and the noise covariance matrix in the Kalman Filter (KF) was estimated. Then, a beamforming algorithm based on KF was developed. To further estimate the Gaussian noise distribution of the received signal phase, the noise covariance matrix was iteratively estimated using unscented transformation (UT), and here, a beamforming algorithm based on the Unscented Kalman Filter (UKF) was proposed. The proposed algorithms were validated through simulations, illustrating their ability to suppress the malign effects of errors on near-field UAV array beamforming. This study provides a reference for the implementation of UAV array beamforming under varying conditions.

## 1. Introduction

As beamforming [1,2,3] and unmanned aerial vehicle (UAV) [4] technology continue to evolve, there has been a rising trend of incorporating antenna array elements equipped with UAVs into distributed electromagnetic wave beamforming systems. It is aimed at achieving directional high-gain signal transmission and enhancing the combat capability of UAVs in challenging environments [5,6]. Although there is limited literature in this field, the distributed characteristic of UAV antenna arrays renders them analogous to MIMO antenna arrays, distributed antenna arrays, and large-phased antenna arrays [7,8,9], thus inspiring research on UAV antenna array technology. The literature [10] highlights that the near-field distance R and maximal antenna aperture L are related to the wavelength λ of the signal, denoted as $R\leq 2L^{2}/\lambda$. Due to the restrictions of UAV spacing, array antenna aperture, etc., the targets of UAVs are mostly located in the near-field area relative to the antenna array composed of UAVs. As a result, conventional far-field antenna electromagnetic field theory does not apply in this case. For instance, an antenna array composed of dozens of UAVs with an aperture of tens of meters will usually have its near-field electronic countermeasure target positioned dozens of kilometers away from the array. Consequently, the application of near-field beamforming technology is critical for improving the quality of the electronic countermeasure mission of UAVs. Near-field beamforming technology has been studied intensively in terahertz (THz) communication conditions [11,12,13,14], but array element error, which is an important factor affecting beamforming performance, has not been considered.

In the presence of UAV position errors, navigation sensor measurement errors, and phase sensor measurement errors in the received signals, beamforming performance is adversely affected [15,16,17,18]. The received signal errors in UAV arrays primarily include array position errors and signal phase sensor measurement errors. The array position errors include errors between the actual position and the ideal position of the array and the navigation sensor measurement error. The navigation data include the position information of the array provided by the navigation position sensor. The literature [19] highlights that in order to achieve effective beamforming, the spatial position accuracy of UAVs should be within half a wavelength of the received or transmitted signal. However, due to errors, the navigation data used to obtain the spatial position accuracy of UAVs frequently fall outside of this range. Kalman Filters (KF), Unscented Kalman Filters (UKF), and Extended Kalman Filters (EKF) are commonly used to achieve better parameter estimation in linear and nonlinear estimation systems, respectively [20,21,22,23,24,25,26,27,28,29,30,31,32,33,34,35,36]. KF-based parameter selection can greatly influence the estimation performance [37,38,39]. One study [40] proposed that filter parameters such as the initial guess, observation noise covariance, and initial estimate error covariance in Kalman-filter-based image reconstruction greatly affect the performance of the method. Inaccurate parameter selection can lead to problems such as a lower convergence rate, artifacts, or filter divergence. Another study [41] demonstrated that the performance of KF deteriorates when the system noise statistics are not available a priori, in which the adjustment of the measurement noise covariance is deemed paramount, as it directly affects the estimation accuracy and plays a crucial role in applications such as sensor selection and sensor fusion.

After conducting a thorough analysis, it was observed that the distributed antenna of UAVs cannot effectively perform beamforming due to the near-field location of the targets as well as the UAVs’ position, time synchronization, and signal phase measurement errors. To address this issue, our team proposes three distributed beamforming algorithms. In Section 2, the construction and analysis of the array’s received signal phase error model and signal processing process are analyzed. Two algorithms are proposed for beamforming based on the EKF and KF under the condition of the presence or absence of navigation data. The third beamforming algorithm, based on the UKF, is proposed for scenarios where the lower sidelobe level is a key requirement. In Section 3, the three algorithms’ processes, based on the EKF, KF, and UKF, respectively, are outlined in detail. In Section 4, the proposed algorithms are verified through simulations, which proves their effectiveness in beamforming. In Section 5, we offer concluding remarks and discuss the selection of algorithms based on specific requirements.

The novelty of our work lies in several aspects: 1. In contrast to the existing works, we innovatively consider the UAV as a sensor and take its errors into consideration under near-field conditions. The UAV array is viewed as a distributed antenna array for which a near-field signal model is constructed, taking into account the UAV position error, measurement error, and time synchronization error. 2. To minimize the UAVs’ error influence on array beamforming, a KF-based beamforming algorithm is proposed using navigation data. The noise covariance is deduced through Taylor expansion on the near-field signal model to ensure the optimal performance of the KF. 3. Due to the possible error induced by Taylor expansion in the KF-based algorithm, a UKF-based beamforming algorithm that provides a more precise estimation of noise distribution compared to the KF-based algorithm, albeit at the cost of an increased computation time, is proposed. 4. An EKF-based beamforming algorithm is proposed for navigation interference scenes, wherein the signal phase is estimated and compensated for based on the UAV’s position estimated using the EKF when navigation data are not available. Although the performance of the EKF-based algorithm is not as effective as that of the previous two algorithms, it can be utilized as a supplementary beamforming method in conditions where navigation data are unobtainable. Overall, three different beamforming algorithms are proposed under different conditions, providing a reference for UAV array application in electronic countermeasure (ECM).

## 2. Model and Analysis of Phase Error in Beamforming

In near-field conditions, the state of an electromagnetic wave propagating in space as a plane wave is deemed impractical, as a spherical waveform is necessary to effectively depict the wave’s propagation in space. Considering that the appraisal of the UAV configuration arranged in a uniform linear array lays the groundwork for further research on other arrangements, our primary focus is placed on the beamforming of UAVs arranged in a uniform, linear array. Because of UAVs’ space limitation, the UAV array is sparse within a large range of possibilities, which may lead to ambiguous beam problems while offering a high-resolution beam advantage [42]. To simplify the scene, we assume that the target is within the main lobe range of the beam.

**Figure 1:**
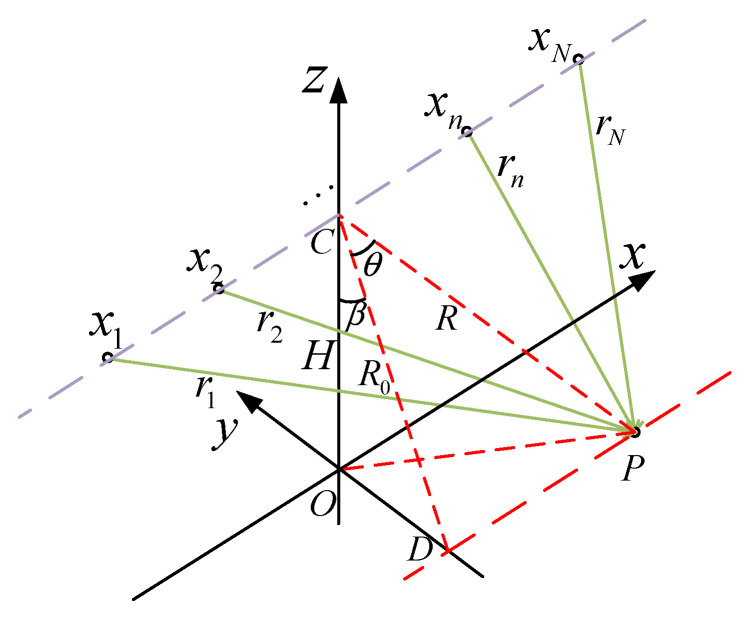
Geometric model of near-field UAV array.

As shown in Figure 1, the *N* elements of the near-field antenna array formed by *N* UAVs are distributed at equal intervals along the axis parallel to the *x*-axis at the height *H*. The total length of the UAV array is L along the purple line in Figure 1. The ideal position coordinate of the *n*th UAV is $\left(x_{n},y_{n},z_{n}\right)$, where n=1,2,⋯,N, $y_{n}=0$, $z_{n}=H$, and $-L/2\leq x_{n}\leq L/2$. The actual position coordinate is $\left({\tilde{x}}_{n},{\tilde{y}}_{n},{\tilde{z}}_{n}\right)$. *P*, located in the xOy plane, denotes the target radiation source of the UAV array in the near-field space, whose coordinate is $P\left(x_{P},y_{P},0\right)$. C denotes the ideal center point of the array. D denotes the projection of P on the *y*-axis. R denotes the distance between P and C. $R_{0}$, shown in red line in Figure 1, denotes the distance between C and D. θ denotes the included angle between line *CP* and *CD*. β denotes the included angle between line *CD* and *CO*. $R_{0}=R\cos\theta$, $x_{P}=R\sin\theta$, $y_{P}=R_{0}\sin\beta$, and $H=R_{0}\cos\beta$ are included. $r_{n}$ denotes the ideal distance between the *n*th array element and P, which can be expressed as $r_{n}=\sqrt{{\left(x_{n}-x_{P}\right)}^{2}+y_{P}^{2}+H^{2}}$ shown in green lines in Figure 1. $\Delta r_{n}$ denotes the difference between the distance from the *n*th array element’s ideal position to P and the length of R shown in red line in Figure 1, which can be expressed as $\Delta r_{n}=r_{n}-R$. ${\tilde{r}}_{n}$ denotes the actual distance between the *n*th array element and P, which can be expressed as ${\tilde{r}}_{n}=\sqrt{{\left({\tilde{x}}_{n}-x_{P}\right)}^{2}+{\left({\tilde{y}}_{n}-y_{P}\right)}^{2}+{\tilde{z}}_{n}^{2}}$. $\Delta {\tilde{r}}_{n}$ denotes the difference between the actual distance and ideal distance from the *n*th array element to P, which can be expressed as $\Delta {\tilde{r}}_{n}={\tilde{r}}_{n}-r_{1}$. One should note that UAVs are small-sized, the UAV’s ideal position is where the UAV is supposed to be, the UAV’s actual position is where it actually is, and the actual signal received by the UAV is what the sensor on the UAV actually receives.

For the radiation source *P*, the actual radiation signal received at the central point C of the array can be expressed as $\tilde{A}=A\exp\left(j\phi_{n0}\right)$, where j represents an imaginary unit and $\phi_{n0}$ represents the signal phase. By taking $\tilde{A}$ as the reference signal, the actual radiation signal received by the *n*th array element can be expressed as [43]:

(1) $$s_{n}=\tilde{A}\exp\left(j\left(\phi_{n}+{\tilde{\phi }}_{Pn}+\Delta \phi_{n}\right)\right)=A\exp\left[j\left(\phi_{n0}+\phi_{n}+{\tilde{\phi }}_{Pn}+\Delta \phi_{n}\right)\right]$$

where $\phi_{n}$ represents the phase difference caused by $\Delta r_{n}$, and it can be expressed as $\phi_{n}=-k\Delta r_{n}$, where k=2π/λ represents the wave number. $\Delta \phi_{n}$ represents the phase synchronization error caused by the time synchronization error between the *n*th UAV and the reference UAV. ${\tilde{\phi }}_{Pn}$ represents the phase difference caused by $\Delta {\tilde{r}}_{n}$, which can be expressed as:

(2) $${\tilde{\phi }}_{Pn}=-k\Delta {\tilde{r}}_{n}=-k\left(\sqrt{{\left({\tilde{x}}_{n}-x_{P}\right)}^{2}+{\left({\tilde{y}}_{n}-y_{P}\right)}^{2}+{\tilde{z}}_{n}^{2}}-r_{n}\right)$$

Then, the true signal phase value ${\tilde{\varphi }}_{n}$ can be expressed as

(3) $${\tilde{\varphi }}_{n}=\phi_{n0}+\phi_{n}+{\tilde{\phi }}_{Pn}$$

The absence of navigation data presents a challenge to array beamforming in regard to phase errors. These errors arise from the UAV’s positional inaccuracies and inaccuracies in the phase measurements of received signals. For the *m*th (*m* = 1, 2, …, *M*) sample, the measurement of the *n*th array element’s received signal phase can be obtained from

(4) $${\hat{\varphi }}_{n,m}=\phi_{n0}+\phi_{n}+{\tilde{\phi }}_{Pn}+\Delta \phi_{n}+\Delta \phi_{n,m}$$

where $\Delta \phi_{n,m}$ represents the phase measurement noise of the *n*th UAV for the *m*th sample. We can see from (4) that the phase synchronization error can be regarded as the measurement error. Substituting Equation (2) into Equation (4), ${\hat{\varphi }}_{n,m}$ can be expressed as

(5) $${\hat{\varphi }}_{n,m}=\phi_{n0}+\phi_{n}+\Delta \phi_{n}+\Delta \phi_{n,m}-k\left(\sqrt{{\left({\tilde{x}}_{n}-x_{P}\right)}^{2}+{\left({\tilde{y}}_{n}-y_{P}\right)}^{2}+{\tilde{z}}_{n}^{2}}-r_{n}\right)$$

It is imperative to complete the phase compensation of the received signal from the intended direction to achieve the desired pointing beam in the array. In the absence of navigation data, one may take the array position as the state variable and the phase measurement as the observation value, thereby using the EKF technique to filter Equation (5) and determine estimates for both the array position and corresponding phase value.

The source of navigation data is typically the navigation sensors, providing crucial positional information for the array. Specifically, this article focuses on the UAV’s spatial position along the *x*, *y*, and *z* axes, which can be implemented as phase compensation values within the model. With precise navigation data, position compensation allows for a reliable assumption that the UAVs’ positions are adjusted from the actual to the ideal. Conversely, the compensation of inaccurate data suggests that the UAVs’ positions approximate the ideal position yet are subject to potential errors within the navigation data.

Array position errors represent a composition of actual and ideal position errors between array elements and position measurement errors when using navigation data. For the *m*th sample, the phase value transformed by the position measurement of the *n*th array element is ${\hat{\phi }}_{Pn,m}$, which can be expressed as

(6) $$\begin{array}{l} {\hat{\phi }}_{Pn,m}=-k\Delta {\hat{r}}_{n,m}\\ =-k\left(\sqrt{{\left({\hat{x}}_{n,m}-x_{P}\right)}^{2}+{\left({\hat{y}}_{n,m}-y_{P}\right)}^{2}+{\hat{z}}_{n,m}^{2}}-r_{n}\right) \end{array}$$

where $\Delta {\hat{r}}_{n,m}$ represents the difference between the actual distance and the ideal distance from the *n*th array element to source *P* for the *m*th sample, and $\left({\hat{x}}_{n,m},{\hat{y}}_{n,m},{\hat{z}}_{n,m}\right)$ represents the position measurement of the *n*th array element for the *m*th sample and can be expressed as

(7) $$\left[\begin{array}{c} {\hat{x}}_{n,m}\\ {\hat{y}}_{n,m}\\ {\hat{z}}_{n,m} \end{array}\right]=\left[\begin{array}{c} {\tilde{x}}_{n}\\ {\tilde{y}}_{n}\\ {\tilde{z}}_{n} \end{array}\right]+R_{n,m}$$

where $R_{n,m}={\left[\begin{array}{ccc} \Delta x_{n,m} & \Delta y_{n,m} & \Delta z_{n,m} \end{array}\right]}^{T}$ represents the 3×1 dimensional measurement noise of the position measurement sensor of the *n*th UAV for the *m*th sample.

Using the position measurement in Equation (6) to compensate Equation (5), there is a residual phase $\Delta \varphi_{n,m}$ of the *n*th UAV for the *m*th sample, expressed as

(8) $$\begin{array}{l} \Delta \varphi_{n,m}={\hat{\varphi }}_{n,m}-{\hat{\phi }}_{Pn,m}\\ =\phi_{n0}+\phi_{n}+\Delta \phi_{n}+\Delta \phi_{n,m}\\ -k\left(\sqrt{{\left({\tilde{x}}_{n}-x_{P}\right)}^{2}+{\left({\tilde{y}}_{n}-y_{P}\right)}^{2}+{\tilde{z}}_{n}^{2}}-\sqrt{{\left({\tilde{x}}_{n}+\Delta x_{n,m}-x_{P}\right)}^{2}+{\left({\tilde{y}}_{n}+\Delta y_{n,m}-y_{P}\right)}^{2}+{\left({\tilde{z}}_{n}+\Delta z_{n,m}\right)}^{2}}\right) \end{array}$$

According to Equation (8), the position measurements of UAVs are recognized as their actual position values to rectify phase errors resulting from variations between actual positions and ideal positions. However, it is important to note that this compensation process may introduce a degree of position measurement error. Clearly, due to the existence of a phase error caused by the position measurement and phase measurement, beamforming cannot be effectively carried out if the N×1 dimensional weighted vector $W={\left[\begin{array}{cccc} w_{1} & w_{2} & \cdots & w_{N} \end{array}\right]}^{T}$ is obtained based on the ideal position of the array where $w_{n}=\exp\left(-jk\Delta r_{n}\right)$. The residual value $\Delta {\hat{\varphi }}_{n}$ is considered the estimation value of the received residual signal phase of the *n*th UAV. Then, the received signals of the array elements are weighted and summed as the results of array beamforming:

(9) $$Y=W^{H}\hat{X}$$

where ${\left[\cdot \right]}^{H}$ represents the matrix conjugate transpose and $\hat{X}$ represents the estimated value of the array’s received signal, which can be expressed as

(10) $$\hat{X}={\left[\begin{array}{ccc} \exp\left(jk\Delta {\hat{\varphi }}_{1}\right) & \cdots & \exp\left(jk\Delta {\hat{\varphi }}_{N}\right) \end{array}\right]}^{T}$$

Typically, the focus of wireless communication, electronic reconnaissance, and jamming lies within the near-field proximity of the UAV array. However, it is worth noting that the distance between the target of interest and the antenna center exceeds the aperture of the antenna array by a considerable margin. Under these conditions, it can be assumed that R≫L. Then, the Taylor series expansion of ${\hat{\varphi }}_{n,m}$ in Equation (5) at the ideal position of the UAV can be obtained from

(11) $$\begin{array}{l} {\hat{\varphi }}_{n,m}=\phi_{n0}+\phi_{n}+\Delta \phi_{n}+\Delta \phi_{n,m}-k\left(\sqrt{{\left({\tilde{x}}_{n}-x_{P}\right)}^{2}+{\left({\tilde{y}}_{n}-y_{P}\right)}^{2}+{\tilde{z}}_{n}^{2}}-r_{n}\right)\\ \approx \phi_{n0}+\phi_{n}+\Delta \phi_{n}+\Delta \phi_{n,m}-\frac{k}{r_{n}}\left(\Delta {\tilde{x}}_{n}\cdot \left(x_{n}-x_{P}\right)+\Delta {\tilde{y}}_{n}\cdot \left(y_{n}-y_{P}\right)+\Delta {\tilde{z}}_{n}\cdot H\right) \end{array}$$

where $\Delta {\tilde{x}}_{n}$, $\Delta {\tilde{y}}_{n}$, and $\Delta {\tilde{z}}_{n}$ represent the differential values between the actual position values of the *n*th UAV and the corresponding ideal position values, respectively, on the three axes.

Similarly, Taylor expansion is performed on the phase measurement ${\hat{\phi }}_{Pn,m}$ shown in Equation (6) as follows:

(12) $$\begin{array}{l} {\hat{\phi }}_{Pn,m}=-k\Delta {\hat{r}}_{n,m}\\ =-k\left(\sqrt{{\left({\hat{x}}_{n,m}-x_{P}\right)}^{2}+{\left({\hat{y}}_{n,m}-y_{P}\right)}^{2}+{\hat{z}}_{n,m}^{2}}-r_{n}\right)\\ \approx -\frac{k}{r_{n}}\left(\left(\Delta x_{n,m}+\Delta {\tilde{x}}_{n}\right)\cdot \left(x_{n}-x_{P}\right)+\left(\Delta y_{n,m}+\Delta {\tilde{y}}_{n}\right)\cdot \left(y_{n}-y_{P}\right)+\left(\Delta z_{n,m}+\Delta {\tilde{z}}_{n}\right)\cdot H\right) \end{array}$$

Substituting Equations (11) and (12) into Equation (8), the residual value $\Delta \varphi_{n,m}$ of the residual signal phase can be approximated as

(13) $$\Delta \varphi_{n,m}\approx \phi_{n0}+\phi_{n}+\Delta \phi_{n}+\Delta \phi_{n,m}-\frac{k}{r_{n}}\left(\Delta x_{n,m}\cdot \left(x_{n}-x_{P}\right)+\Delta y_{n,m}\cdot \left(y_{n}-y_{P}\right)+\Delta z_{n,m}\cdot H\right)$$

Due to the independence of the position measurement errors across the three axes and their compliance with the Gaussian distribution, Equation (13) suggests that the noise distribution of the residual signal phase can be approximated as Gaussian. As a result, the measurement noise variance in the KF algorithm can be determined. By approximating the distribution of $\Delta \varphi_{n,m}$ as Gaussian, UT can be applied to $\Delta \varphi_{n,m}$ prior to Taylor expansion, and the standard Gaussian distribution parameter can be estimated accordingly. Subsequently, the beamforming algorithm based on the UKF can be implemented. When the near-field signal processing is considered, we can see that our methods provide the phase estimation values that include range information, as shown in Equation (8).

## 3. Parameter Estimation and Beamforming Based on KF, EKF, and UKF

The Taylor expansion approximation of the residual signal phase indicates that the cumulative phase errors resulting from both the position and phase sensor measurement errors conform to an approximately Gaussian distribution. By estimating the estimated noise covariance matrix, a KF-based beamforming algorithm is proposed.

To approximate the probability density distribution based on certain sampling rules, UT is utilized to linearize the function, considering the approximate Gaussian distribution of the residual signal phase. Thus, a UKF-based beamforming algorithm is proposed. By utilizing a series of Sigma points to create a Gaussian distribution, the probability density distribution of the residual signal phase can be approximated. By estimating the residual signal phases, beam formation can be optimized.

Both of the algorithms mentioned earlier require accurate navigation data. When such data are unavailable, it is necessary to develop an alternative solution so as to effectively form a beam. To address this issue, an EKF-based beamforming algorithm is proposed. The EKF is utilized to estimate the array position, as shown in Equation (5), and subsequently filter the array signal phase. This estimation of the array position is then transformed into an estimation of the phase, which is used to compensate for the signal. In order to provide a comprehensive understanding of our proposed approach, the KF-based beamforming algorithm is first described, followed by the UKF-based beamforming algorithm and the EKF-based beamforming algorithm.

### 3.1. KF-Based Beamforming Algorithm

The Kalman Filter is achieved by utilizing the current state measurement to generate a new and more accurate estimation, which is then used to update the error covariance matrix. The steps of KF are shown in Equation (14):

(14) $$\left.\begin{array}{c} \begin{array}{c} g_{m,m-1}=Ag_{m-1}+Bu_{m}\\ P_{m,m-1}=AP_{m-1}A^{T}+Q \end{array}\\ K_{m}=\frac{P_{m,m-1}H^{T}}{HP_{m,m-1}H^{T}+R}\\ g_{m}=g_{m,m-1}+K_{m}\left(m_{m}-Hg_{m,m-1}\right)\\ P_{m}=\left(I-K_{m}H\right)P_{m,m-1} \end{array}\right\}$$

where $g_{m,m-1}$ represents the predicted value of system state $g_{m}$ for the *m*th sample according to the system state $g_{m-1}$ for the (*m* − 1)th sample; A represents the system state transition matrix; $u_{m}$ represents the input vector for the *m*th sample; B represents the system control matrix; $P_{m,m-1}$ represents the predicted value of covariance matrix $P_{m}$ for the *m*th sample according to the covariance matrix $P_{m-1}$ for the (*m* − 1)th sample; Q represents the system noise matrix; R represents the measurement noise matrix; $K_{m}$ represents the Kalman gain matrix for the *m*th sample; $m_{m}$ represents the measurement for the *m*th sample; H represents the measurement matrix; and I represents the identity matrix.

According to Equation (13), the phase difference state equation and measurement equation of UAVs are based on residual measurements of the signal phase, as shown in Equation (15):

(15) $$\left.\begin{array}{c} \phi_{n,m}=A_{n}\cdot \phi_{n,m-1}+n_{n}\\ {\hat{\phi }}_{n,m}=H_{n}\cdot \phi_{n,m}+v_{n} \end{array}\right\}$$

where $A_{n}=1$, $H_{n}=1$, $\phi_{n,m}=\phi_{n0}+\phi_{n}$, ${\hat{\phi }}_{n,m}$ represents the measurement of $\phi_{n,m}$; $n_{n}$ represents the noise caused by the phase state measurement error, which approximately meets the Gaussian distribution with a 0 mean value and $Q_{n}$ covariance; and $v_{n}$ represents the noise caused by the phase measurement errors of the *n*th UAV on the three axes, which approximately meets the Gaussian distribution with a 0 mean value and $R_{n}$ covariance. As stated in the literature [38], $Q_{n}$ and $R_{n}$ directly affect the estimation accuracy, and the performance of the KF will deteriorate if its value is not appropriately set. To solve this problem, we derive $Q_{n}$ and $R_{n}$ from Equation (13) as

(16) $$\left.\begin{array}{c} Q_{n}=k^{2}\left(\frac{R_{x0}\cdot {\left(x_{n}-x_{P}\right)}^{2}+R_{y0}\cdot {\left(y_{n}-y_{P}\right)}^{2}+R_{z0}\cdot H^{2}}{r_{n}^{2}}\right)\\ R_{n}=k^{2}\left(\frac{R_{x}\cdot {\left(x_{n}-x_{P}\right)}^{2}+R_{y}\cdot {\left(y_{n}-y_{P}\right)}^{2}+R_{z}\cdot H^{2}}{r_{n}^{2}}\right)+R_{\phi } \end{array}\right\}$$

where $R_{x0}$, $R_{y0}$, and $R_{z0}$ represent the position transition covariances, respectively, on the *x*, *y*, and *z* axes; $R_{x}$, $R_{y}$, and $R_{z}$ represent the position measurement covariances, respectively, on the *x*, *y*, and *z* axes; and $R_{\phi }$ represents the phase measurement covariance. The KF-based beamforming algorithm described in this paper can be summarized as shown in Algorithm 1.
**Algorithm 1.** KF-based beamforming algorithm.**Inputs:** The total number of elements is *N*, length of the array is *L*, total number of samples is *M*, ideal array position is $\left(x_{n},0,H\right)$, target position is $\left(x_{P},y_{P},0\right)$, measurements of the array position are $\left({\hat{x}}_{n,m},{\hat{y}}_{n,m},{\hat{z}}_{n,m}\right)$ (*n* = 1, 2, …, *N*, *m* = 1, 2, …, *M*), measurements of the array signal phases are ${\hat{\varphi }}_{n,m}$, error variances of the array position measurement on the axes are, respectively, $R_{x}$, $R_{y}$, and $R_{z}$, error variances of the array position transition on the axes are, respectively $R_{x0}$, $R_{y0}$, and $R_{z0}$, the error variance of the phase measurements is $R_{\phi }$. **Outputs:** The estimation values of residual phases are $\Delta \hat{\varphi }$, the values of beamforming are $\tilde{Y}$. **Step 1**: According to the ideal array position and target position, ideal weights are calculated as $W={\left[\begin{array}{cccc} w_{1} & w_{2} & \cdots & w_{N} \end{array}\right]}^{T}$, where $w_{n}=\exp\left(-jk\Delta r_{n}\right)$. According to Equation (16), the state and measurement noise covariances in KF are, respectively, calculated as $Q_{n}$ and $R_{n}$. By substituting the array position measurements $\left({\hat{x}}_{n,m},{\hat{y}}_{n,m},{\hat{z}}_{n,m}\right)$ into Equation (6) and substituting Equation (6) into Equation (5), $\Delta \varphi_{n,m}$ is obtained using Equation (8). **Step 2**: According to Equation (14), KF is performed. For the *n*th element:➀Initialize parameters. The first estimation value of KF is $g_{n,1}=\Delta \varphi_{n,1}$.➁Calculate $g_{n,m,m-1}=g_{n,m}$.➂Calculate $P_{n,m,m-1}=P_{n,m-1}+Q_{n}$.➃Calculate $K_{n,m}=\frac{P_{n,m,m-1}}{P_{n,m,m-1}+R_{n}}$.➄Calculate $g_{n,m}=g_{n,m,m-1}+K_{n,m}\left(\Delta \varphi_{n,m}-g_{n,m,m-1}\right)$.➅Calculate $P_{n,m}=\left(1-K_{n,m}\right)P_{n,m,m-1}$.
**Step 3**: Estimation values of the residual phase are obtained from $\Delta \hat{\varphi }=\left[\begin{array}{cccc} \Delta {\hat{\varphi }}_{1} & \Delta {\hat{\varphi }}_{2} & \cdots & \Delta {\hat{\varphi }}_{N} \end{array}\right]$. **Step 4**: Estimation values of the beamforming are obtained from $\tilde{Y}=W^{H}\hat{X}=A\sum_{n=1}^{N}\left(w_{n}^{*}\cdot \exp\left(jk\Delta {\hat{\varphi }}_{n}\right)\right)$.

### 3.2. UKF-Based Beamforming Algorithm

The steps of UKF are as follows.

1. Initialize. In this process, we can obtain $\Delta \varphi_{n,1}$ as the first estimation value of our UKF beamforming algorithm.
2. UT is used in the state function to update the Sigma point set:

(17) $$\left.\begin{array}{c} \chi_{i,m-1}={\hat{x}}_{m-1}\begin{array}{cc} , & i=0 \end{array}\\ \chi_{i,m-1}={\hat{x}}_{m-1}+{\left(\sqrt{\left(k+\rho \right)P_{m-1}}\right)}_{i},\;i=1,\cdots ,k\\ \chi_{i,m-1}={\hat{x}}_{m-1}-{\left(\sqrt{\left(k+\rho \right)P_{m-1}}\right)}_{i},\;i=k+1,\cdots ,2k \end{array}\right\}$$

where *k* represents the state dimension, the total number of Sigma sampling points is 2k+1, $\hat{x}$ represents the mean value of the sampling points, P represents the state error covariance matrix, $\chi_{i}$ represents the *i*th group of Sigma sampling points, and ρ represents the scaling ratio, which is used to reduce the total prediction error.

3. The mean value and covariance of the target state are predicted according to the Sigma point set:

(18) $$\left.\begin{array}{c} {\hat{x}}_{m\left|m-1\right.}=\sum_{i=0}^{2k}\omega_{i}\chi_{i,m\left|m-1\right.}\\ P_{m\left|m-1\right.}=\sum_{i=0}^{2k}\omega_{i}\left(\chi_{i,m\left|m-1\right.}-{\hat{x}}_{m\left|m-1\right.}\right){\left(\chi_{i,m\left|m-1\right.}-{\hat{x}}_{m\left|m-1\right.}\right)}^{T}+Q \end{array}\right\}$$

where $\left.m\right|m-1$ represents the value in the *m*th snapshot according to the (*m* − 1)th snapshot and $\omega_{i}$ represents the weight of the *i*th sampling point.

4. The measurement transfer function is again processed using UT, and the measurement prediction is updated to generate new Sigma points as follows:

(19) $$\left.\begin{array}{c} \zeta_{i,m\left|m-1\right.}={\hat{x}}_{m\left|m-1\right.}\begin{array}{cc} , & i=0 \end{array}\\ \zeta_{i,m\left|m-1\right.}={\hat{x}}_{m\left|m-1\right.}+{\left(\sqrt{\left(k+\rho \right)P_{m\left|m-1\right.}}\right)}_{i}\begin{array}{cc} , & i=1,\cdots ,k \end{array}\\ \zeta_{i,m\left|m-1\right.}={\hat{x}}_{m\left|m-1\right.}-{\left(\sqrt{\left(k+\rho \right)P_{m\left|m-1\right.}}\right)}_{i}\begin{array}{cc} , & i=k+1,\cdots ,2k \end{array} \end{array}\right\}$$

5. The mean value, covariance, and cross-covariance of the measurements are predicted according to the new Sigma point set:

(20) $$\left.\begin{array}{c} {\overset{⌢}{m}}_{m\left|m-1\right.}=\sum_{i=0}^{2k}\omega_{i}m_{i,m\left|m-1\right.}\\ P_{zz,m\left|m-1\right.}=\sum_{i=0}^{2k}\omega_{i}\left(m_{i,m\left|m-1\right.}-{\overset{⌢}{m}}_{m\left|m-1\right.}\right){\left(m_{i,m\left|m-1\right.}-{\overset{⌢}{m}}_{m\left|m-1\right.}\right)}^{T}+R\\ P_{xz,m\left|m-1\right.}=\sum_{i=0}^{2k}\omega_{i}\left(\zeta_{i,m\left|m-1\right.}-{\hat{x}}_{m\left|m-1\right.}\right){\left(m_{i,m\left|m-1\right.}-{\overset{⌢}{m}}_{m\left|m-1\right.}\right)}^{T}+R \end{array}\right\}$$

where $m_{i,m\left|m-1\right.}$ represents the system measurement corresponding to the new Sigma points for the *m*th sample.

6. State correction. The Kalman gain is calculated, and the state variance is updated:

(21) $$\left.\begin{array}{c} K_{m}=P_{xz,m\left|m-1\right.}P_{zz,m\left|m-1\right.}^{-1}\\ {\hat{x}}_{m}={\hat{x}}_{m\left|m-1\right.}+K_{m}\left({\hat{m}}_{m}-{\overset{⌢}{m}}_{m\left|m-1\right.}\right)\\ P_{m}=P_{m\left|m-1\right.}-K_{m}P_{zz,m\left|m-1\right.}K_{m}^{T} \end{array}\right\}$$

In this work, we posit that the UAVs execute the ECM mission while hovering. Considering that the UAV’s state transition demonstrates linearity, the sole task required is to undertake a measurement using UT, followed by the execution of the KF technique. Based on the UKF, the beamforming algorithm is summarized in Algorithm 2.
**Algorithm 2.** UKF-based beamforming algorithm.**Inputs:** The total number of elements is *N*, length of the array is *L*, total number of samples is *M*, ideal array position is $\left(x_{n},0,H\right)$, target position is $\left(x_{P},y_{P},0\right)$, measurements of the array position are $\left({\hat{x}}_{n,m},{\hat{y}}_{n,m},{\hat{z}}_{n,m}\right)$ (*n* = 1, 2, …, *N*, *m* = 1, 2, …, *M*), measurements of the array signal phases are ${\hat{\varphi }}_{n,m}$, error variances of the array position measurements on the axes are, $R_{x}$, $R_{y}$, and $R_{z}$, respectively, the error variance of the phase measurements is $R_{\phi }$, state dimension is *k*, total number of Sigma points is 2*k* (*i* = 1, 2, …, 2*k*), scaling ratio is ρ, the weights of the sampling points are ω. **Outputs:** The estimation values of the residual phases are $\Delta \hat{\varphi }$, the values of beamforming are $\tilde{Y}$. **Step 1**: According to the ideal array position and target position, ideal weights are calculated as $W={\left[\begin{array}{cccc} w_{1} & w_{2} & \cdots & w_{N} \end{array}\right]}^{T}$, where $w_{n}=\exp\left(-jk\Delta r_{n}\right)$. According to Equation (16), the initial noise covariance in UKF is calculated as $P_{0}$. By substituting the array position measurements $\left({\hat{x}}_{n,m},{\hat{y}}_{n,m},{\hat{z}}_{n,m}\right)$ into Equation (6) and substituting Equation (6) into Equation (5), $\Delta \varphi_{n,m}$ is obtained using Equation (8). **Step 2**: UKF is performed for the *n*th element.➀Initialize parameters. The first estimation value of UKF is $\Delta \varphi_{n,1}$, the noise covariance is $P_{n,0}=1$;➁UT is performed, and $\chi_{n,i,ss-1}$ is obtained using Equation (17).➂Mean value and covariance of the Sigma points are obtained from ${\hat{x}}_{n,m\left|m-1\right.}=\sum_{i=0}^{2k}\omega_{n,i}\chi_{n,i,m-1}$ and $P_{n,m\left|m-1\right.}=\sum_{i=0}^{2k}\omega_{n,i}\left(\chi_{n,i,m-1}-{\hat{x}}_{n,m\left|m-1\right.}\right){\left(\chi_{n,i,m-1}-{\hat{x}}_{n,m\left|m-1\right.}\right)}^{T}$, as shown in Equation (20).➃KF is performed as shown in Equation (21), and the Kalman gain is obtained from $K_{n,m}=\frac{P_{n,m\left|m-1\right.}}{P_{n,m\left|m-1\right.}+P_{n,0}}$.➄Calculate ${\hat{x}}_{n,m}={\hat{x}}_{n,m\left|m-1\right.}+K_{n,m}\left(\Delta \varphi_{n,m}-{\hat{x}}_{n,m\left|m-1\right.}\right)$.➅Calculate $P_{n,m}=\left(1-K_{n,m}\right)P_{n,m\left|m-1\right.}$. **Step 3**: Estimation values of the residual phase are obtained from $\Delta \hat{\varphi }=\left[\begin{array}{cccc} \Delta {\hat{\varphi }}_{1} & \Delta {\hat{\varphi }}_{2} & \cdots & \Delta {\hat{\varphi }}_{N} \end{array}\right]$. **Step 4**: Estimation values of beamforming are obtained from $\tilde{Y}=W^{H}\hat{X}=A\sum_{n=1}^{N}\left(w_{n}^{*}\cdot \exp\left(jk\Delta {\hat{\varphi }}_{n}\right)\right)$.

### 3.3. EKF-Based Beamforming Algorithm

The state equation and measurement equation of the EKF algorithm can be expressed as:

(22) $$\left.\begin{array}{c} g_{m}=f\left(g_{m-1},n_{m}\right)\\ m_{m}=h\left(g_{m},v_{m}\right) \end{array}\right\}$$

where $f\left(\cdot \right)$ and $h\left(\cdot \right)$ represent the second-order differentiable equations, respectively, of the state and measurement equations. After linearization, we obtain

(23) $$\left.\begin{array}{c} g_{m}=f\left(g_{m-1}\right)+F_{m-1}\left(g_{m-1}-g_{m-2}\right)+n_{m}\\ m_{m}=h\left(g_{m}\right)+H_{m}\left(g_{m}-g_{m-1}\right)+v_{m} \end{array}\right\}$$

where $F_{m}={\left.\frac{\partial f}{\partial g}\right|}_{{\hat{g}}_{m-1}}$, $H_{m}={\left.\frac{\partial h}{\partial g}\right|}_{g_{m}}$, $N_{m}={\left.\frac{\partial f}{\partial n}\right|}_{g_{m-1}}$, $V_{m}={\left.\frac{\partial h}{\partial v}\right|}_{g_{m,m-1}}$.

The primary distinction between the EKF and KF is that when the state equation and measurement equation are nonlinear systems, the Jacobian matrix corresponding to either equation changes for every sample based on the discrepancy between the estimated value and the predicted value, as demonstrated in Equation (24):

(24) $$\left.\begin{array}{c} \begin{array}{c} g_{m,m-1}=f\left(g_{m-1},0\right)\\ P_{m,m-1}=F_{m}P_{m-1}F_{m}^{T}+N_{m}QN_{m}^{T} \end{array}\\ K_{m}=\frac{P_{m,m-1}H_{m}^{T}}{H_{m}P_{m,m-1}H_{m}^{T}+V_{m}RV_{m}^{T}}\\ g_{m}=g_{m,m-1}+K_{m}\left(m_{m}-h\left(g_{m,m-1},0\right)\right)\\ P_{m}=\left(I-K_{m}H_{m}\right)P_{m,m-1} \end{array}\right\}$$

The proposed phase model is processed using the EKF when the UAV performs its ECM task. By utilizing the residual value of the signal phase as described in Equation (5), the state transition function can be simplified as a linear function, with a first-order Taylor expansion performed for the measurement function. Firstly, the actual position of the array is estimated based on the signal received from the desired direction. The position estimation is then transformed into a phase value for the phase compensation value. From Equation (5), we can obtain Equation (25) as follows:

(25) $$\begin{array}{ccc} g_{n,m}=\left[\begin{array}{c} x_{n,m}-x_{P}\\ y_{n,m}-y_{P}\\ z_{n,m}-z_{P} \end{array}\right]=A_{n,m}\cdot \left[\begin{array}{c} x_{n,m-1}-x_{P}\\ y_{n,m-1}-y_{P}\\ z_{n,m-1}-z_{P} \end{array}\right]+\left[\begin{array}{c} n_{n,x,m}\\ n_{n,y,m}\\ n_{n,z,m} \end{array}\right],\; & \begin{array}{l} m_{n,m}=h\left(g_{n,m,m-1},v_{n,m}\right)\\ =\phi_{n0}+v_{n,m}\\ -k\left(\sqrt{{\left(x_{n,m}^{}-x_{P}\right)}^{2}+{\left(y_{n,m}^{}-y_{P}\right)}^{2}+{\left(z_{n,m}^{}-z_{P}\right)}^{2}}-R\right) \end{array}, & H_{ss}=\left[\begin{array}{c} \frac{-k\left(x_{n,m}^{}-x_{P}\right)}{\sqrt{{\left(x_{n,m}^{}-x_{P}\right)}^{2}+{\left(y_{n,m}^{}-y_{P}\right)}^{2}+{\left(z_{n,ss}^{}-z_{P}\right)}^{2}}}\\ \frac{-k\left(y_{n,m}^{}-y_{P}\right)}{\sqrt{{\left(x_{n,m}^{}-x_{P}\right)}^{2}+{\left(y_{n,m}^{}-y_{P}\right)}^{2}+{\left(z_{n,m}^{}-z_{P}\right)}^{2}}}\\ \frac{-k\left(z_{n,m}^{}-z_{P}\right)}{\sqrt{{\left(x_{n,m}^{}-x_{P}\right)}^{2}+{\left(y_{n,m}^{}-y_{P}\right)}^{2}+{\left(z_{n,m}^{}-z_{P}\right)}^{2}}} \end{array}\right] \end{array}$$

where $A_{n,m}$ represents the third-order identity matrix, and $x_{n,m}$, $y_{n,m}$, and $z_{n,m}$ represent the position estimation on the three axes of the *n*th UAV, respectively, for the *m*th sample. The EKF-based beamforming algorithm discussed in this paper can be summarized as shown in Algorithm 3.
**Algorithm 3.** EKF-based beamforming algorithm.**Inputs:** The total number of elements is *N*, length of the array is *L*, total number of samples is *M*, ideal array position is $\left(x_{n},0,H\right)$, target position is $\left(x_{P},y_{P},0\right)$, measurements of the array signal phases are ${\hat{\varphi }}_{n,m}$, (*n* = 1, 2, …, *N*, *m* = 1, 2, …, *M*), error variances of the array position measurements on the axes are $R_{x}$, $R_{y}$, and $R_{z}$, respectively, the error variance of the phase measurements is $R_{\phi }$. **Outputs:** The estimation values of signal phases are $\hat{\varphi }$, the values of beamforming are $\tilde{Y}$. **Step 1**: According to the ideal array position and target position, ideal weights are calculated as $W={\left[\begin{array}{cccc} w_{1} & w_{2} & \cdots & w_{N} \end{array}\right]}^{T}$, where $w_{n}=\exp\left(-jk\Delta r_{n}\right)$. According to Equation (24), $F_{n,m}=\left[\begin{array}{ccc} 1 & 0 & 0\\ 0 & 1 & 0\\ 0 & 0 & 1 \end{array}\right]$, $N_{n,m}=\left[\begin{array}{ccc} 1 & 0 & 0\\ 0 & 1 & 0\\ 0 & 0 & 1 \end{array}\right]$, the initial noise covariance is $Q=\left[\begin{array}{ccc} R_{x} & 0 & 0\\ 0 & R_{y} & 0\\ 0 & 0 & R_{z} \end{array}\right]$, initial value of cross covariance is $P_{1}=\left[\begin{array}{ccc} 1 & 0 & 0\\ 0 & 1 & 0\\ 0 & 0 & 1 \end{array}\right]$, covariance of measurement noise is $R=R_{\phi }$, $V_{n,m}=1$, and $H_{n,m}$, as shown in Equation (25). **Step 2**: According to Equation (24), when the target signal of the desired direction is received, EKF is performed to estimate the phase difference caused by the range difference between the actual array position and ideal array position. ➀Initialize parameters. The first estimation value of state is $g_{n,1}={\left[\left(x_{n}-x_{P}\right),\left(y_{n}-y_{P}\right),\left(z_{n}-z_{P}\right)\right]}^{T}$, and the first filter value of EKF is $h\left(g_{n,1},0\right)=\phi_{n0}-k\left(\sqrt{{\left(x_{n}^{}-x_{P}\right)}^{2}+{\left(y_{n}^{}-y_{P}\right)}^{2}+{\left(z_{n}^{}-z_{P}\right)}^{2}}-R\right)$.➁Calculate $g_{n,m,m-1}=g_{n,m-1}$, $P_{n,m,m-1}=P_{n,m-1}$.➂Calculate $h\left(g_{n,m,m-1},0\right)$.➃Calculate $K_{n,m}=\frac{P_{n,m,m-1}H_{n,m}^{T}}{H_{n,m}P_{n,m,m-1}H_{n,m}^{T}+R_{n}}$.➄Calculate $g_{n,m}=g_{n,m,m-1}+K_{n,m}\left({\hat{\varphi }}_{n,m}-h\left(g_{n,m,m-1},0\right)\right)$.➅Calculate $P_{n,m}=\left(I-K_{n,m}H_{n,m}\right)P_{n,m,m-1}$. **Step 3**: The position difference between the actual array position and target position is obtained from $\hat{g}=\left[\begin{array}{cccc} {\hat{g}}_{1} & {\hat{g}}_{2} & \cdots & {\hat{g}}_{N} \end{array}\right]$, which can be transformed to a phase value as a phase compensation function using ${\hat{\varphi }}_{P}=k\left[\begin{array}{cccc} {\left\|{\hat{g}}_{1}\right\|}_{2}-r_{1} & {\left\|{\hat{g}}_{2}\right\|}_{2}-r_{2} & \cdots & {\left\|{\hat{g}}_{N}\right\|}_{2}-r_{N} \end{array}\right]$, where ${\left\|\cdot \right\|}_{2}$ represents 2-Norm. **Step 4**: According to **Step 2**, estimation values of the signal phase are obtained from $\hat{\varphi }=\left[\begin{array}{cccc} {\hat{\varphi }}_{1} & {\hat{\varphi }}_{2} & \cdots & {\hat{\varphi }}_{N} \end{array}\right]$. **Step 5**: By compensating $\hat{\varphi }$ with ${\hat{\varphi }}_{P}$, estimation values of the residual phase are obtained from $\Delta \hat{\varphi }=\left[\begin{array}{cccc} \Delta {\hat{\varphi }}_{1} & \Delta {\hat{\varphi }}_{2} & \cdots & \Delta {\hat{\varphi }}_{N} \end{array}\right]$. **Step 6**: Estimation values of beamforming are obtained from $\tilde{Y}=W^{H}\hat{X}=A\sum_{n=1}^{N}\left(w_{n}^{*}\cdot \exp\left(jk\Delta {\hat{\varphi }}_{n}\right)\right)$.

### 3.4. Performance Analysis for the Proposed Algorithms

In the KF-based beamforming algorithm, UAV navigation data are used to compensate for the received signal phase. Therefore, the KF-based beamforming algorithm is suitable for scenarios where UAV navigation data can be obtained. Because there is matrix inversion in the KF, the complexity of the classical KF is *O*($d^{3}$), where *d* represents state variable dimensions. In our case, the three-dimensional position is transformed into the one-dimensional phase that serves as our state variable. Thus, the level of complexity in our algorithm is mainly determined by the number of array elements and snapshots involved. According to Algorithm 1, the signals received from the *N* array elements each have *M* snapshots for the desired target position. For every array element, there are five steps involved in the KF. Therefore, ignoring the additive constant term, the computational complexity of the KF-based beamforming algorithm is *O*(5*NM*).

The UKF-based beamforming algorithm is also suitable for scenarios where UAV navigation data can be obtained. Because the noise distribution of the UKF-based beamforming algorithm is more precisely estimated in contrast to that of the KF-based beamforming algorithm, the beam performance of the former is better. According to Algorithm 2, for the *N* elements, a UT transformation is performed first. The computational complexity for the *k* variables is *O*(2*N* + 4*Nk*). Then, the UKF with a computational complexity of *O*(6*NMk* + 5*NM*) is performed. Overall, ignoring the additive constant term, the computational complexity of the algorithm is *O*(2*N* + 4*Nk* + 5*NM* + 6*NMk*). For the model in this paper, with the UT transformation variable *k* = 1, the complexity of the UKF beamforming algorithm is simplified to *O*(6*N* + 11*NM*).

In the EKF-based beamforming algorithm, the UAV position is estimated by using the EKF algorithm and transformed into signal phase estimation, indicating that it is suitable for scenarios lacking UAV navigation data. According to Algorithm 3, the true positions of *N* array elements are first estimated using EKF based on the received signal phase, and its computational complexity is *O*(3*N* + 12*NM*). Then, the position estimation of the array elements is converted into signal phase estimation, and the signal phase is filtered again with a computational complexity of *O*(3*N* + 12*NM*). Ignoring the additive constant term, the total computational complexity is *O*(6*N* + 24*NM*).

## 4. Simulation Analysis

### 4.1. Parameter Estimation Simulation

**Figure 2:**
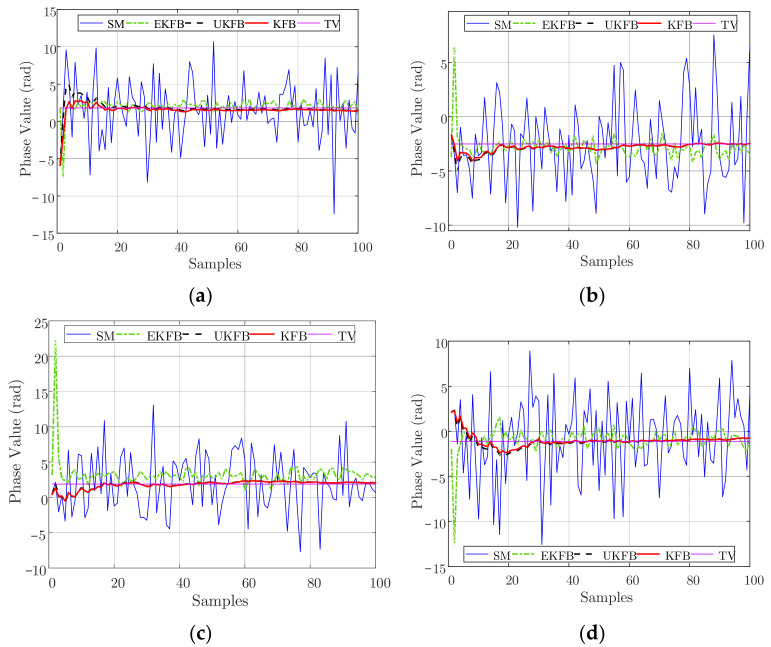
When $\sigma_{R}=0.4\;m$, the phase estimation of a UAV is as follows: (**a**) $\sigma_{X}=0.2\;\mathrm{rad}$, (**b**) $\sigma_{X}=0.4\;\mathrm{rad}$, (**c**) $\sigma_{X}=0.6\;\mathrm{rad}$, (**d**) $\sigma_{X}=0.8\;\mathrm{rad}$.

**Figure 3:**
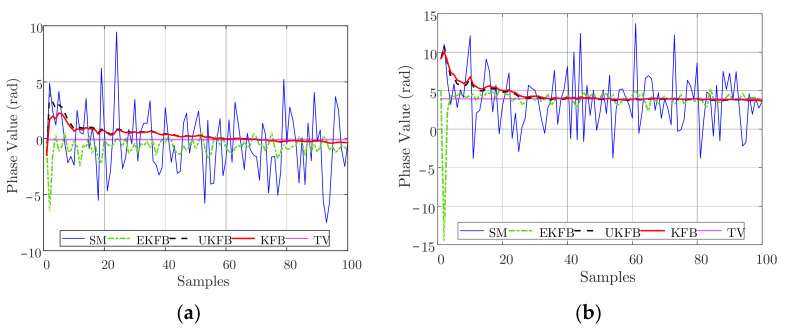

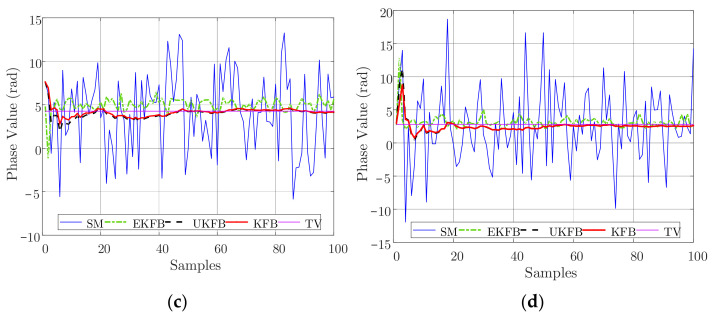
When $\sigma_{X}=0.4\;\mathrm{rad}$, the phase estimation of a UAV is as follows: (**a**) $\sigma_{R}=0.2\;m$, (**b**) $\sigma_{R}=0.4\;m$, (**c**) $\sigma_{R}=0.6\;m$, (**d**) $\sigma_{R}=0.8\;m$.

We suppose that the signal frequency is $f_{0}=300\;\mathrm{MHz}$, the wavelength is λ=1 m, the total number of array elements is N=26, the array aperture is L=500λ, and the ideal height of the array elements is H=1000 m, $R_{0}=30\;\mathrm{km}$, θ=0° or θ=6°, and $R=R_{0}/\cos\theta$. Furthermore, the ideal position of the array is an equally spaced linear distribution along the axis parallel to the *x*-axis at the height *H* and symmetrical to the *y*-axis; the main lobe of the beam points to a known radiation source $P\left(x_{P},y_{P},0\right)$, where $x_{P}=R\sin\theta$ and $y_{P}=R_{0}\sin\beta$; the total number of signal samples is M=100; the position measurement errors of the UAV on the *x*, *y*, and *z* axes all follow Gaussian distributions with a 0 mean value and $\sigma_{R}$ covariance, where $\sigma_{R}$ is changeable from 0.2 m to 0.8 m with an interval of 0.2 m. The phase measurement error follows a Gaussian distribution with a 0 mean value and $\sigma_{X}$ covariance, where $\sigma_{X}$ is changeable from 0.2 rad to 0.8 rad with an interval of 0.2 rad. In the UKF, the state dimension is *k* = 1, and the scaling ratio is ρ=6. References [44,45] introduce an envelope synchronization combining a two-way time comparison algorithm based on UAVs, and the time synchronization accuracy of this algorithm is less than 0.1 ns. Reference [46] described a technique for determining the true time delay using the ambiguous two-tone matched filter output and obtained a timing precision of 2.26 ps. Thus, we assume that the time synchronization between the UAVs is estimated as Δt=0.1 ns, and its phase difference is $\Delta \varphi =\frac{2\pi c\Delta t}{\lambda }\approx 0.1884\;\mathrm{rad}<\frac{\pi }{8}\;\mathrm{rad}$, which indicates that the synchronization of frequency meets our beamforming requirements. All data are generated through simulations. Simulated measurements are denoted as SM. Ture values are denoted as TV. The estimation values were calculated, respectively, using the KFB, EKFB, and UKFB algorithms, denoted as KFB, EKFB, and UKFB. The initial values of the algorithms are consistent with the first SMs. In beamforming, the array generally scans and searches for signals within a certain spatial range. According to the expected direction of arrival, the array selects the corresponding weighted value to add weight to the incoming signals received by the array. When there is a signal in the expected direction, the array will form a main lobe oriented toward that direction. Taking the estimation of the beamforming parameters when there is a signal from the θ=0° direction as an example, when $\sigma_{R}=0.4\;m$, the KFB, EKFB, and UKFB algorithms are used to estimate the signal phase, respectively, and the phase estimation values for every sample are obtained as shown in Figure 2. When $\sigma_{X}=0.4\;\mathrm{rad}$, the KFB, EKFB, and UKFB algorithms are used to estimate the signal phase, respectively, and the phase estimation values for every sample are obtained, as shown in Figure 3.

Based on Figure 2 and Figure 3, both the KFB and UKFB algorithms can converge to the true value within a few dozen samples. It is noteworthy that the KFB algorithm exhibits a smaller jitter amplitude for the estimated value when compared to the UKFB algorithm. This is primarily due to the fact that the function described in this paper can be approximated as a linear function, allowing for a superior estimation outcome with the KFB algorithm. Conversely, the UKFB algorithm utilizes Sigma points to approximate the probability density function, necessitating a significantly longer time to calculate the estimation results. Regarding the EKFB algorithm, its performance is heavily dependent on the SM values because of the poor nonlinear characteristic of the UAV hovering model.

**Figure 4:**
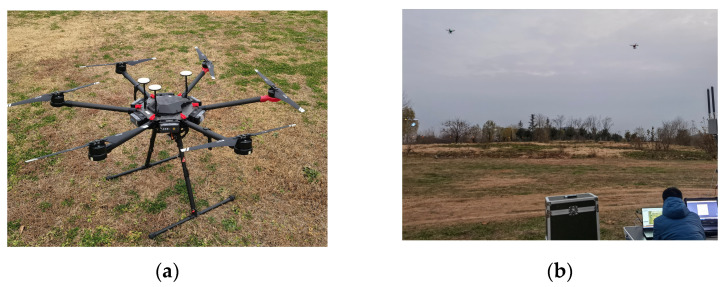

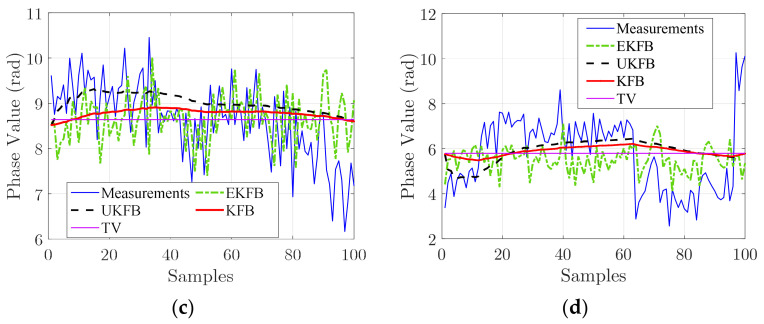
Data collection and phase estimation: (**a**) DJI UAV platforms, (**b**) experimental scene, (**c**) phase estimation of the 5th UAV, (**d**) phase estimation of the 11th UAV.

To further verify the efficiency of our algorithms, we performed some experiments with hovering DJI UAV platforms, as shown in Figure 4a,b. In the experiments, the position measurement errors of DJI UAVs were gathered. Using these data, the measurement data illustrated in Figure 4c,d were generated by combining the position measurement errors of the UAVs with the simulated phase measurement errors. Here, the covariance $\sigma_{X}$ of the simulated phase measurement errors was set to 0.4 rad, and the other parameters remained consistent with those used in the simulation settings. The resulting phase estimations are illustrated in Figure 4c,d.

**Figure 5:**
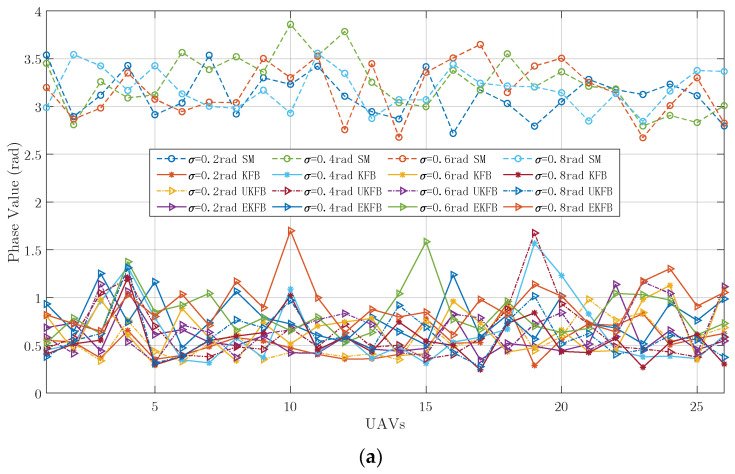

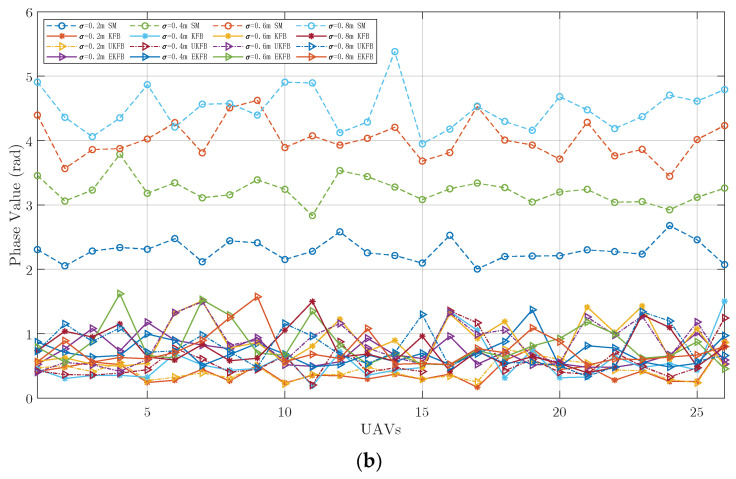
Array phase estimation error: (**a**) $\sigma_{R}=0.4\;m$, (**b**) $\sigma_{X}=0.4\;\mathrm{rad}$.

Analyzing Figure 4, we can observe that the outcomes are comparable to those presented in Figure 2 and Figure 3, indicating the efficiency of our algorithms. Furthermore, Figure 5 shows the average estimated phase errors for all the array elements across all samples.

The results in Figure 5 indicate that, on the one hand, both the KFB and UKFB algorithms exhibit comparable low-level phase estimation errors. On the other hand, the EKFB algorithm exhibits slightly higher phase estimation errors. Notably, when the phase error $\sigma_{X}$ changes from 0.2 rad to 0.8 rad under $\sigma_{R}=0.4\;m$, the estimation errors of all three algorithms only exhibit minor changes. When the phase error $\sigma_{R}$ changes from 0.2 m to 0.8 m under $\sigma_{X}=0.4\;\mathrm{rad}$, the estimation errors of the KFB and UKFB algorithms exhibit minor changes, while the estimation errors of the SM and EKFB algorithms in the phase domain change significantly. This phenomenon can be attributed to the influence of the wave number, where the position errors of the array elements are amplified, leading to larger SM phase errors. Nevertheless, the KFB and UKFB algorithms exhibit greater robustness in combating error influence. Furthermore, although SM errors impact the performance of the EKFB algorithm, its estimation error is much smaller than the SM error. Notably, compared to the UKFB algorithm, the KFB algorithm is much simpler but exhibits a similar or slightly poorer phase estimation performance, which underscores the former’s advantages.

### 4.2. Beamforming Simulation

**Figure 6:**
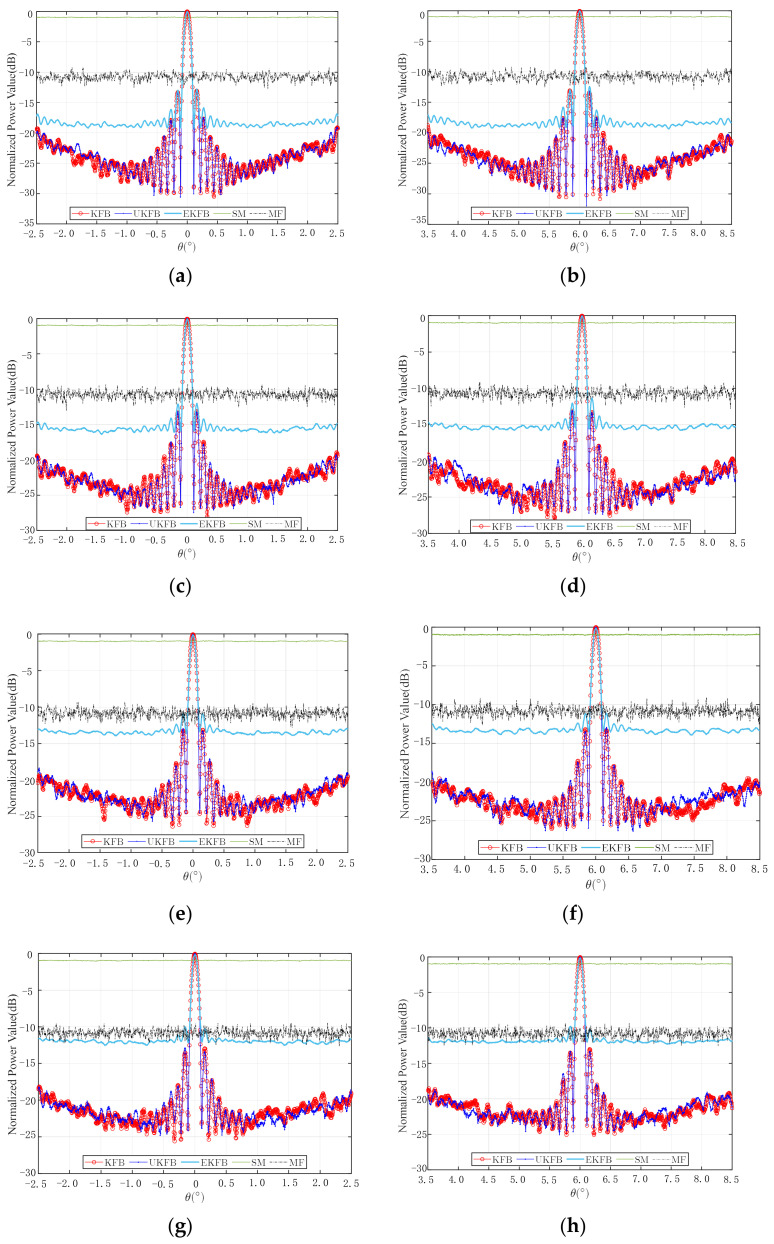
Beamforming: (**a**) θ=0°, $\sigma_{R}=0.2\;m$, $\sigma_{X}=0.2\;\mathrm{rad}$, (**b**) θ=6°, $\sigma_{R}=0.2\;m$, $\sigma_{X}=0.2\;\mathrm{rad}$, (**c**) θ=0°, $\sigma_{R}=0.4\;m$, $\sigma_{X}=0.4\;\mathrm{rad}$, (**d**) θ=6°, $\sigma_{R}=0.4\;m$, $\sigma_{X}=0.4\;\mathrm{rad}$, (**e**) θ=0°, $\sigma_{R}=0.6\;m$, $\sigma_{X}=0.6\;\mathrm{rad}$, (**f**) θ=6°, $\sigma_{R}=0.6\;m$, $\sigma_{X}=0.6\;\mathrm{rad}$, (**g**) θ=0°, $\sigma_{R}=0.8\;m$, $\sigma_{X}=0.8\;\mathrm{rad}$, (**h**) θ=6°, $\sigma_{R}=0.8\;m$, $\sigma_{X}=0.8\;\mathrm{rad}$.

This section compares the three algorithms based on their time, main lobe width, and the average sidelobe level of the beams formed. The simulation parameters remain unchanged, as mentioned in Section 4.1. To ensure comparability, a Median Filter is added as a filter commonly used to estimate the phase values of UAVs. Values estimated using a Median Filter [47] are denoted as MF. The beamforming is then obtained by conducting 100 Monte Carlo simulations of each algorithm, as shown in Figure 6. The average sidelobe level is defined as the mean value of sidelobe values below −10 dB, and the main lobe width is the half-power beam width. The average time, average sidelobe level, and main lobe width of each algorithm are presented in Table 1.

The results presented in Figure 6 and Table 1 indicate that when using the SM or MF for phase compensation, one cannot form a beam, regardless of whether the beam is scanning or not. Additionally, the average sidelobe level of the EKFB algorithm is significantly impacted by SM errors, as the phase estimation values exhibit considerable jitter. As a result, it is difficult for the EKFB algorithm to converge to the true value within a 100-sample period, leading to uncertainty in the final phase estimation value due to the influence of the SM. As the SM errors increase, the uncertainty and error in the phase compensation value also increase. Under most conditions, the beam sidelobe level of the UKFB algorithm is slightly lower than that of the KFB algorithm, while the average time of the KFB algorithm is consistently the shortest. It is important to note that it may take the UKFB algorithm up to 15 times longer and the EKFB algorithm up to 30 times longer to run than the KFB algorithm, primarily due to the extensive calculation requirements associated with Sigma points simulation or measurement function Jacobian matrix estimation. Additionally, when SM errors increase, the sidelobe levels of the beams formed by the KFB and UKFB algorithms may also slightly increase. Nevertheless, within the range of SM errors examined in this paper, all three algorithms can effectively form a beam, underscoring the robustness of the KFB and UKFB algorithms to SM errors.

**Figure 7:**
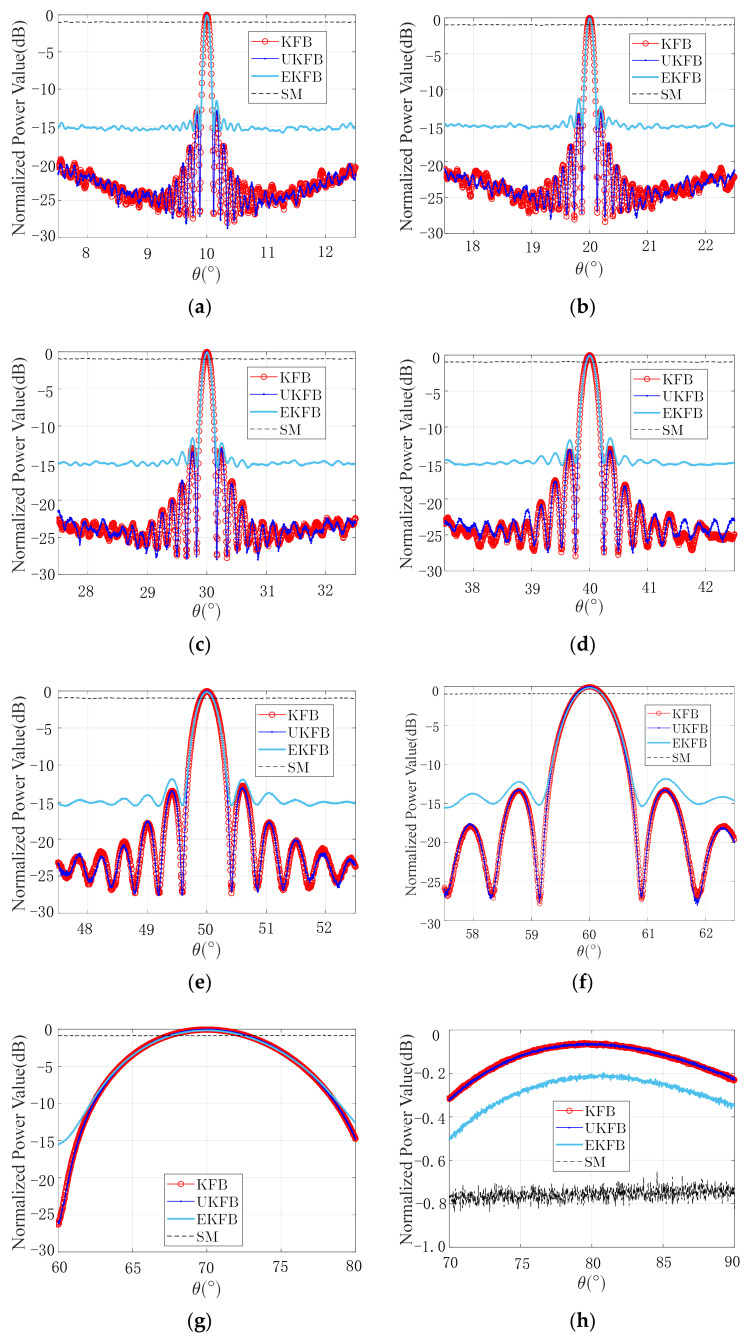
Beam scans: (**a**) θ=10°, (**b**) θ=20°, (**c**) θ=30°, (**d**) θ=40°, (**e**) θ=50°, (**f**) θ=60°, (**g**) θ=70°, (**h**) θ=80°.

When the beam scanning range is large enough, the beamforming pattern will change significantly. When the other simulation conditions remain unchanged, $\sigma_{R}=0.4\;m$ and $\sigma_{X}=0.4\;\mathrm{rad}$, and the expected direction of the angle changes to θ=10°:10°:80°. The beamforming is shown in Figure 7.

As depicted in Figure 7, the KFB, UKFB, and EKFB algorithms are capable of effectively forming a beam with a greater main lobe width as the direction of the angle increases to θ=10°:10°:70°. This is due to the decreasing difference in the distance caused by the same angle range, which results in a slower change in the phase difference of the array received signal and, subsequently, a slower change in the final composite beam power [1]. The qualities of beams generated by the KFB and UKFB algorithms are comparable. The KFB algorithm demonstrates a shorter algorithm time of approximately 0.2 s compared to the 3.3 s of the UKFB algorithm, rendering the former algorithm more suitable for meeting real-time beamforming requirements. In instances where the desired beam angle is excessively large, such as θ=80°, depicted in Figure 7h, none of the algorithms can form a beam. However, it is worth noting that the desired angle is not commonly observed in real cases. Utilizing navigation data, while the UKFB algorithm produces a slightly lower sidelobe level compared to the KFB algorithm, the beam generated by the latter satisfies both the lower sidelobe level and real-time requirements. In the absence of navigation data, the EKFB algorithm can effectively form a beam. In the practical ECM application of UAVs, these simulation results can be applied to control the array scanning angle, allowing one to opt for an appropriate beamforming algorithm based on the task requirements.

## 5. Conclusions

In this article, we propose three distributed beamforming algorithms that overcome the problem of ineffective performance caused by the position and signal measurement errors through a distributed array of UAVs. Initially, we addressed the array’s received signal phase error model and, without navigation data, introduced a beamforming algorithm based on the EKF. In addition, we proposed beamforming algorithms based on the KF and UKF for navigation data utilization. The effectiveness of the proposed algorithms was verified via simulation, indicating their ability to perform array beamforming. The simulation results demonstrate that the proposed algorithms can suppress the malign effect of errors on near-field array beamforming, offering new methods for ECM application in UAV arrays. It is recommended that the EKF-based algorithm be utilized when navigation data are unavailable, while the KF-based algorithm is recommended for higher real-time and lower sidelobe levels. In contrast, the UKF-based algorithm is suitable for situations where real-time requirements are not high, but there is a dire need for a relatively lower sidelobe level. This study focused on ECM tasks carried out by hovering UAVs. In the future, we will consider the flight path and motion of UAVs and explore the possibility of reducing the greater computational complexity of the Sigma point method to improve the real-time beamforming capability of the UKF-based algorithm.

## Figures and Tables

**Table 1:** Algorithm performance comparison table.

	Index	$\sigma_{R}$$,\;\sigma_{X}$ Value	θ=0°			θ=6°		
Algorithm			Average Time of Algorithm (s)	Average Sidelobe Level (dB)	Main Lobe Width (°)	Average Time of Algorithm (s)	Average Sidelobe Level (dB)	Main Lobe Width (°)
KFB		$\sigma_{R}=0.2\;m$ $\sigma_{X}=0.2\;\mathrm{rad}$	0.2703	−23.8775	0.094	0.2698	−24.0005	0.0970
UKFB			3.2502	−23.9203	0.094	3.2922	−24.0247	0.0980
EKFB			8.6538	−18.2883	0.094	7.1471	−18.2838	0.0910
KFB		$\sigma_{R}=0.4\;m$ $\sigma_{X}=0.4\;\mathrm{rad}$	0.2650	−22.9399	0.092	0.2696	−22.8134	0.0980
UKFB			3.2373	−22.9773	0.092	3.2888	−22.9625	0.0980
EKFB			7.2024	−15.5946	0.092	7.1468	−15.3128	0.0980
KFB		$\sigma_{R}=0.6\;m$ $\sigma_{X}=0.6\;\mathrm{rad}$	0.2655	−22.1120	0.0985	0.2686	−22.1552	0.0980
UKFB			3.2394	−21.9564	0.0983	3.2911	−22.1734	0.0983
EKFB			7.2007	−13.6235	0.0983	7.1441	−13.5411	0.0980
KFB		$\sigma_{R}=0.8\;m$ $\sigma_{X}=0.8\;\mathrm{rad}$	0.2644	−21.5081	0.0983	0.2677	−21.5187	0.0996
UKFB			3.2345	−21.5867	0.0982	3.2896	−21.5938	0.0995
EKFB			7.2048	−12.4386	0.0982	7.1452	−12.3304	0.0993

## Data Availability

Not applicable.
